# Detection of* Cryptosporidium* and* Cyclospora *Oocysts from Environmental Water for Drinking and Recreational Activities in Sarawak, Malaysia

**DOI:** 10.1155/2017/4636420

**Published:** 2017-11-06

**Authors:** Lesley Maurice Bilung, Ahmad Syatir Tahar, Nur Emyliana Yunos, Kasing Apun, Yvonne Ai-Lian Lim, Elexson Nillian, Hashimatul Fatma Hashim

**Affiliations:** ^1^Faculty of Resource Science and Technology, Universiti Malaysia Sarawak, 94300 Kota Samarahan, Sarawak, Malaysia; ^2^Department of Parasitology, Faculty of Medicine, University of Malaya, 50603 Kuala Lumpur, Malaysia

## Abstract

Cryptosporidiosis and cyclosporiasis are caused by waterborne coccidian protozoan parasites of the genera* Cryptosporidium* and* Cyclospora,* respectively. This study was conducted to detect* Cryptosporidium* and* Cyclospora* oocysts from environmental water abstracted by drinking water treatment plants and recreational activities in Sarawak, Malaysia. Water samples (12 each) were collected from Sungai Sarawak Kanan in Bau and Sungai Sarawak Kiri in Batu Kitang, respectively. In addition, 6 water samples each were collected from Ranchan Recreational Park and UNIMAS Lake at Universiti Malaysia Sarawak, Kota Samarahan, respectively. Water physicochemical parameters were also recorded. All samples were concentrated by the iron sulfate flocculation method followed by the sucrose floatation technique.* Cryptosporidium* and* Cyclospora* were detected by modified Ziehl-Neelsen technique. Correlation of the parasites distribution with water physicochemical parameters was analysed using bivariate Pearson correlation. Based on the 24 total samples of environmental water abstracted by drinking water treatment plants, all the samples (24/24; 100%) were positive with* Cryptosporidium*, and only 2 samples (2/24; 8.33%) were positive with* Cyclospora*. Based on the 12 total samples of water for recreational activities, 4 samples (4/12; 33%) were positive with* Cryptosporidium*, while 2 samples (2/12; 17%) were positive with* Cyclospora*.* Cryptosporidium* oocysts were negatively correlated with dissolved oxygen (DO).

## 1. Introduction

*Cryptosporidium* and* Cyclospora* are coccidian protozoan parasites that are the causative agents of waterborne outbreaks worldwide with faecal oral route as the infection transmission.* Cryptosporidium* is one of the leading pathogens which are responsible for majority diarrhoeal infections [1]. There are two most common species infecting human, namely,* Cryptosporidium hominis* and* Cryptosporidium parvum*. Infectious dose of the parasite varies upon human immune status. A study on human volunteers revealed that the median infectious dose of* C. parvum* (ID_50_) infection is 132 oocysts for healthy individuals and as low as 30 oocysts can initiate an infection [2]. A person without previous exposure to cryptosporidiosis is more susceptible to low dose of oocyst as no anti-*C. parvum*-specific immunoglobulins is found in the body [3].

*Cryptosporidium* oocyst has become a concern for the water industry as it is infectious, robust in the environment, and resistant to disinfectants (chlorine and chloramines) and can compromise filter bed of the water filtration system [4, 5]. In other developed countries such as the United States and Canada,* Cryptosporidium* represents one of the key parameters for determining the safety of environmental water as drinking water supply [6, 7] and was classified under Category 1 in Unitary Environmental Classification of Water- and Excreta-Related Disease.

*Cyclospora cayetanensis* can also cause prolonged diarrhoea, nausea, and abdominal cramps, and human is the only natural host of the parasite [4]. The parasite is resistant to chlorination like* Cryptosporidium*. Medications usually given to treat enteric diseases such as albendazole, azithromycin, norfloxacin, tinidazole, quinacrine, nalidixic acid, and diloxanide furoate are not effective against* Cyclospora* [8]. Infectious dose of* Cyclospora* is unknown but suggested to be as low as 10–100 oocysts [9].

Survivability of* Cryptosporidium* in drinking water has drawn substantial concern by the water and health agencies. To the best of our knowledge, only two published studies about* Cryptosporidium* contamination on raw water for water treatment plants have been conducted in Malaysia. These have revealed occurrence of* Cryptosporidium* to be within 0–0.06 oocyst/L [10, 11]. Although there is no outbreak of cryptosporidiosis up to the present time in the country, many sporadic cases have been reported in immunocompromised individuals [12–17] and animals [18–23]. These do not count the undiagnosed and asymptomatic individuals, self-limiting cases, and unpublished data. In other Asian countries, the parasite has been encountered in tap water as little as one oocyst from 115 samples [24]. In Northern Thailand, 14.42% of natural river water samples (15/104) were contaminated with the parasite [25], while a study on untreated and treated water from 20 frozen food factories in Thailand found 35% untreated water samples (7/20) were positive with the parasite but 0% in treated water samples (0/20) [26].

Sungai Sarawak Kanan, Bau and Sungai Sarawak Kiri, and Batu Kitang are rivers contributing to important water basins. The raw waters are abstracted by the nearby water treatment plants to supply treated water to the areas of Bau and Kuching [27]. Ranchan Recreational Park is 60 km away from Kuching which is located at the south of Serian. It gains much attraction from the locals especially during weekend. UNIMAS Lake is a man-made lake located at the west wing of the main campus of Universiti Malaysia Sarawak and is popular among students for kayaking activity. Previously, Richard et al. [11] conducted a study on parasite contamination on Sungai Sarawak Kiri. However, the present study will also include Sungai Sarawak Kanan as sources of river water abstracted by drinking water treatment plants. This study is the first to determine the contamination of* Cryptosporidium* and* Cyclospora* in Sarawak recreational areas. The finding of this study provides preliminary data to illustrate parasite occurrences in the study areas.

## 2. Materials and Methods

### 2.1. Sampling Sites and Collection

A total of 24 raw water samples for drinking were collected from Sungai Sarawak Kanan in Bau (*n* = 12) and Sungai Sarawak Kiri in Batu Kitang (*n* = 12), while a total of 12 water samples for recreational activities were collected from Ranchan Recreational Park (*n* = 6) and UNIMAS Lake (*n* = 6). The samples of raw water for drinking were collected separately at surface and subsurface (approximately 5 meters depth) at each station (upstream, midstream, and downstream) from Sungai Sarawak Kanan and Sungai Sarawak Kiri. The water was collected by pumping into sterile polypropylene containers with the aid of a vacuum pump (Rhos Motor) using 12 V energy source from a car battery. The distance between each sampling station was approximately between 500 and 2000 meters due to poor accessibility. The water samples for recreational activities were collected at the surface by using the similar equipment. The distance between each sampling station was approximately 100 meters. The samples of both for drinking and recreational were collected once in two weeks constitutively, starting from January 2017 to March 2017.

### 2.2. Measurements of Physicochemical Parameters

The physicochemical parameters analysed in this study were temperature, pH (Walklab pH meter, TI9000), conductivity, Total Dissolved Solid (Cyberscan meter, CON II), turbidity (Martini, Mi 415), and dissolved oxygen (Professional Series oxygen meter, YSI Pro 20). All the physicochemical parameters were measured in the lab except for temperature and pH. The data were recorded.

### 2.3. Flocculation and Sedimentation

This method was in accordance with the procedure by Karanis and Kimura [28] with little modification. Firstly, 20 ml of ferric sulfate (0.2525 M) solution was added to 10 l of the water samples and the pH was adjusted to 6.0 ± 0.05. Flocs would form and were let to settle overnight (approximately 14 h) at room temperature. Afterwards, the clear fluid was cautiously discarded without disturbing the sediment. The sediments were centrifuged at 2,100 ×g for 10 min (4°C, no brake) and supernatant was discarded until leaving approximately 1 ml of pellet and added with 1 ml of lysis buffer (0.3997 M citric acid monohydrate, 0.5998 M trisodium citrate dihydrate, pH 4.7). The pellets were incubated at room temperature for 1 h (with intermittent vortexing every 15 min). Subsequently, the lysis buffer was washed off by centrifugation at 2,100 ×g for 10 min (4°C) with distilled water up to 50 ml. The washing step was performed twice.

### 2.4. Sucrose Flotation

This procedure was in accordance with the procedure by Kuczynska & Shelton [29] with a little modification with the increment of specific gravity. The pellets were underlaid with sucrose solution (3.7362 M, 1.27 specific gravity) and centrifuged at 300 ×g for 5 minutes. A coverslip was gently placed on the top of the negative meniscus of the samples and let to stand for 30 minutes. The samples attached on the coverslip surface were scrapped into microcentrifuge tubes and washed thrice at 1000 ×g for 10 minutes. The final pellets were concentrated to 50 *μ*l volume with distilled water.

### 2.5. Detection of* Cryptosporidium* and* Cyclospora* Oocyst

A volume of 50 *μ*l of the samples was stained with modified Ziehl-Neelsen technique, according to Casemore et al. [30]. The samples were observed under a microscope at 1000x magnification and measured by using Cell^∧^D software (Olympus).* Cryptosporidium* oocysts appeared as pinkish red, almost spherical and measured 4–6*μ*m [31].* Cyclospora* oocysts appeared light clear pink to deep red, containing granules or bubbly appearance, and measured 8–10 *μ*m [32]. The samples were observed twice to prevent errors. The results were compared with the image gallery by Centers for Disease Control and Prevention [33].

### 2.6. Statistical Analysis

The concentration of* Cryptosporidium *and* Cyclospora *oocysts were expressed per litre by dividing the number of positive respective parasites with ten litres. Correlation between the parasites occurrence and physicochemical parameters was analysed by using bivariate Pearson correlation analysis. The data were analysed using SPSS software version 24.0 (IBM, New York). The distribution of the oocyst based on streams and water column was expressed in mean.

## 3. Results

Overall, there were a total of 36 environmental water samples collected, comprised of 24 river water samples used for drinking and 12 river and lake water samples used for recreational activities. As displayed in Tables 1 and 4, 77.8% (*n* = 28/36) of these water samples were positive for* Cryptosporidium* oocysts, whereas 11.1% (*n* = 4/36) were found to be contaminated with* Cyclospora.*

### 3.1. Occurrence of* Cryptosporidium* and* Cyclospora* in Environmental Water Samples Abstracted by Drinking Water Treatment Plants

Of the 24 environmental water samples (i.e., river) abstracted by drinking water treatment plants, 58.3% (14/24) were positive with* Cryptosporidium* oocysts. Higher concentrations were detected in water samples from Sungai Sarawak Kiri compared to Sungai Sarawak Kanan. Only 8.33% (2/24) were positive with* Cyclospora. *These positive samples were from Sungai Sarawak Kiri. All the samples from Sungai Sarawak Kanan were negative (Table 1).

**Figure 1:**
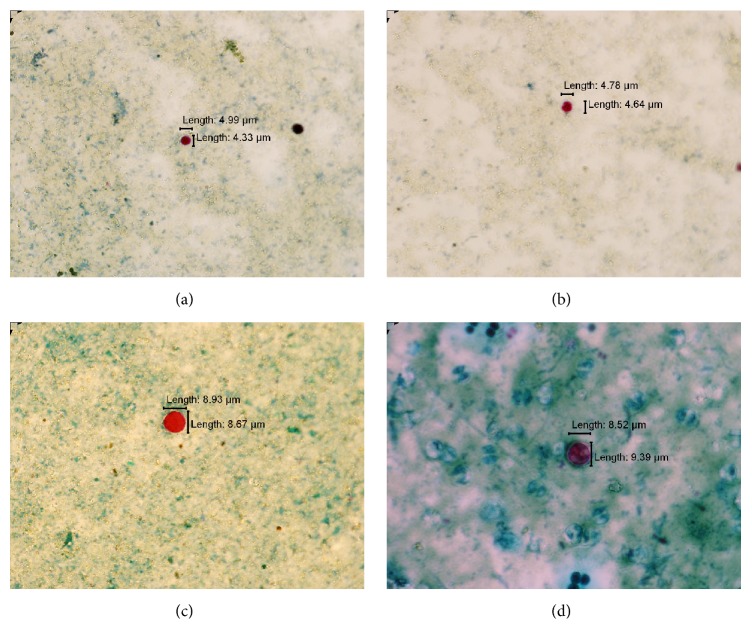
((a) and (b))* Cryptosporidium* oocyst detected in subsurface water samples from Sungai Sarawak Kiri, (c)* Cyclospora* oocyst detected in a surface water sample from Sungai Sarawak Kiri, and (d)* Cyclospora* oocyst detected in a water sample from UNIMAS Lake.

In addition, distribution of* Cryptosporidium *and* Cyclospora* oocysts was also analysed based on the sampling site of the river system (i.e., upstream, midstream and downstream) and water column (i.e., surface and subsurface). As shown in Table 2, the highest number of* Cryptosporidium* and* Cyclospora* was found at the downstream sampling sites. Refer to Figure 1 for the representatives of* Cryptosporidium* and* Cyclospora* oocysts detected.

Higher concentrations of* Cryptosporidium* oocyst were detected from the subsurface compared to the surface. In contrast, concentrations of* Cyclospora* oocyst were higher in water samples from the surface compared to the subsurface (Table 3).

### 3.2. Occurrence of* Cryptosporidium* and* Cyclospora* in Environmental Water Samples Used for Recreational Activities

Out of the total 12 samples collected from UNIMAS Lake and Ranchan Recreational Park, 33.3% (4/12) were positive with* Cryptosporidium.* All the positive samples were isolated from UNIMAS Lake. No* Cryptosporidium* was found in the samples from Ranchan Recreational Park. As for* Cyclospora*, 16.7% (2/12) were positive in water samples from UNIMAS Lake. No* Cyclospora* was detected in the samples from Ranchan Recreational Lake (Table 4).

### 3.3. Correlation of the Parasite Oocyst Occurrence with Physicochemical Parameters

Based on Table 5, distribution of* Cryptosporidium* oocysts had no correlation with all the physicochemical parameters except DO with negative correlation. Correlation of* Cyclospora *and the physicochemical parameters was not analysed in this study due to the small number of positive samples.

## 4. Discussion

*Cryptosporidium* (18/36; 50%) were detected more than* Cyclospora *(4/36; 11.11%) from both types of water used for drinking and recreational activities. In comparison,* Cryptosporidium* is of greater public health concerns as it has been the causative agent for numerous waterborne outbreaks due to its small size (4–6 *μ*m) and being infectious after being shed from the infected hosts.* Cyclospora *is usually shed in low numbers even by immunocompromised hosts. Outbreaks of cryptosporidiosis have no seasonality compared to cyclosporiasis. As this pattern,* Cryptosporidium* can cause higher number of infections than* Cyclospora*. These might be the reasons of* Cyclospora* being scarce in the water samples [34].

### 4.1. Occurrence of* Cryptosporidium* and* Cyclospora* in Environmental Water Samples Abstracted by Drinking Water Treatment Plants

There were a higher number of positive samples with* Cryptosporidium* (14/24) than* Cyclospora* (2/24) from Sungai Sarawak Kanan and Sungai Sarawak Kiri.* Cryptosporidium* were detected in the range of 0.1–2.7 oocysts/L, while* Cyclospora* were detected in the range of 0.1–0.2 oocysts/L. Both rivers contribute to the raw water supply to Bau Water Treatment Plant and Batu Kitang Water Treatment Plant. Both parasites have monoxenous development, low dose of oocysts, and resistant to most disinfectants [35, 36], but* Cryptosporidium* is more hazardous because of being readily infectious after shed [37]. The occurrence of these waterborne parasites is of public health concern for the water treatment industries. High occurrence of these parasites in natural water can lead to an outbreak, whereas low numbers of oocysts may not be detected during surveillance.

The present study obtained higher concentrations of* Cryptosporidium* (0.1–2.7 oocysts/L) compared to the study by Richard et al. [11] (0.02–0.04 oocysts/L) from raw water of Sungai Sarawak Kiri. This might be due to the different concentration methods used. The current study applied flocculation which in some reported recorded better recovery rates compared to membrane filtration method [38–40].

The water treatment industries in Malaysia rely on conventional treatment processes which include coagulation, flocculation, sedimentation, filtration, disinfection, and pH adjustment. Alum-lime and chloramination are used during coagulation and disinfection, respectively [27]. According to a report, nearly all water treatment plants practising this system are at risk of passing* Cryptosporidium* into public treated water at the rate of 52 infections per 10,000 people per year [41]. The worst outbreak ever recorded took place in Milwaukee in 1993. The principal causes were attributed to poor quality of natural water supply and faulty flocculation and filtration processes. These vents subsequently led to increase of turbidity and* Cryptosporidium* in the treated water [42].

*Cryptosporidium* is one of the most resistant and recalcitrant pathogens in water. Its oocyst can withstand high concentration chlorine treatment for 18 hours and chloramines. Advanced studies have revealed that chlorine oxide, ozone, and ultraviolet (UV) can inactivate* Cryptosporidium*, but several advantages such as scale-up issues and turbidity interference impede extrapolation in the real circumstances [43]. Suboptimal flocculation and filtration allow the oocysts to survive into the drinking water supply [44]. Size of* Cryptosporidium* which is one-third of an amoeba or* Giardia *can compromise the filtration barrier [5]. Installation of high throughput filtration system such as bank filtration and membrane filtration is capable of filtering out* Cryptosporidium* from the treated water [41] but these technologies are costly.

Cryptosporidiosis is more severe to acquired or congenital immunocompromised individuals such as children particularly below 5 years old, elders, and people with chronic illnesses like Human Immunodeficiency Virus (HIV) infection [12–17, 45]. Public awareness about the practice of boiling water is crucial to prevent further infection in households.* Cryptosporidium* can be inactivated by boiling at 72.4°C or higher or at 64.2°C at least for 2 minutes [46]. Alternatively, water purifiers with 1 *μ*m filter can be used [47, 48]. The infection is riskier to animals through contaminated drinking water. The most common species is* Cryptosporidium parvum* that infects neonatal calves particularly aged 1–3 weeks [49].

The present results showed that* Cryptosporidium* oocysts were the highest in water samples from the downstream (0.71 ± 0.94 oocysts/L) of the rivers. This is followed by the midstream (0.25 ± 0.23 oocysts/L) and the upstream (0.22 ± 0.29 oocysts/L).* Cyclospora* were only present from the downstream, but the quantity was very low (0.03 ± 0.07 oocysts/L). The downstream areas receive water from upstream and midstream. Any contaminations from the two areas can contaminate downstream as well. Besides, this study suggests that contaminations of* Cryptosporidium* and* Cyclospora* in the areas most probably originated from the housing areas, animal farms, waste disposal, and swimming activities along the river.

Both Sungai Sarawak Kanan and Sungai Sarawak Kiri have settlement areas at the downstream sampling site. Contamination from the lands can be brought to the rivers by drainage or water runoff during raining. Animal farms were seen adjacent to midstream area.* C. parvum* is the most encountered species infecting cattle besides* C. muris* with lower incidence reported in prevalence studies. It has been reported that infected cattle sheds high load and frequency of the parasite oocyst in faeces. High contamination can happen from the farms when no barriers or buffer zones such as vegetation areas are set up or if herds are not thwarted from roaming near to the stream [50]. Another possible factor of contamination could be by the unhygienic waste disposal such as nappies which were observed in Sungai Sarawak Kiri. Some bathers were also seen swimming during the sampling days. It must be noted that any person experiencing diarrhoea should avoid getting in contact with water to prevent contamination with* Cryptosporidium* [51].

No distinct difference was noted in the distribution of* Cryptosporidium* from surface (0.391 ± 0.347 oocysts/L) and subsurface (0.4 ± 0.804 oocysts/L) water column. A study has highlighted that oocyst resuspension can occur at surface and subsurface of the water column influenced by various factors such as rainfall intensity, river flow [52], water usage, human activities, and effluent discharge flowrate. Specific gravity of* Cryptosporidium* is 1.080 s.p. [53] and 1.05–1.31 *μ*m/s of settling velocity [54]. Although information on depth of Sungai Sarawak Kanan and Sungai Sarawak Kiri is not available, available information stated that the duration of days taken by parasite oocyst to settle to 5-meter subsurface is approximately 55 days 2 hours–44 days 4 hours and the settling rate would increase if oocysts are attached to particulate matters and subjected to the velocity of the matters [25, 55].

Higher concentrations of* Cyclospora* were found from surface water samples (0.016 ± 0.057 oocysts/L) than subsurface (0.008 ± 0.028 oocysts/L) of the water column. This pattern could suggest that the introduction of this parasite into the water has recently occurred and the oocysts float freely with the water flow. Although information of its settling velocity is not available, it can be postulated that the settling velocity would probably be faster than* Cryptosporidium* due to its bigger size.

### 4.2. Occurrence of* Cryptosporidium* and* Cyclospora* in Environmental Water Samples Used for Recreational Activities

A higher number of samples were positive with* Cryptosporidium* (4/12) than* Cyclospora* (2/12) from UNIMAS Lake and Ranchan Recreational Park.* Cryptosporidium* were detected in the range of 0.2–1.2 oocysts/L, while* Cyclospora* were detected in the range of 0.2–0.6 oocysts/L. This study did not detect* Cryptosporidium* and* Cyclospora* at all the 6 samples from Ranchan Recreational Park. The fast streamflow might have swept away parasites in the water lead to underreporting especially if the oocysts were present in low numbers. Sunderland et al. [56] pointed out that there was a strong correlation with the number of bathers and presence of* Cryptosporidium*. The public health concern still exists in Ranchan as its waterfall receives many visitors especially during weekends and holidays where contamination from swimmers can happen as mentioned.

In UNIMAS Lake, only* Cryptosporidium* oocysts were detected which were all from inlet areas that receive drainage water from hostels and restaurants. However, information on the status of water quality of the drainage water from the hostels and restaurants is not available. However, the risk of contracting cryptosporidiosis is still low because the students mainly do kayaking during curricular activities. High risk can happen through oral transmission such as accidental swallowing of the water when the students fall into the water.

To reduce water contamination by swimming activities, the public should (i) reduce the number of bathers; (ii) restrict children with diapers from being near to water; and (iii) impede bathers with gastrointestinal diseases from swimming; (iv) bathers should use shower before swimming; (v) swimming areas should be far from sources of contaminations [57].

### 4.3. Correlation of* Cryptosporidium* and* Cyclospora* with Physicochemical Parameters

Physicochemical parameters are preliminary indicator of water quality to indicate oocysts distribution. Among the 6 physicochemical parameters,* Cryptosporidium* was negatively correlated with DO (*p* < 0.01). Based on the previous studies, it is noteworthy that* Cryptosporidium* had association with turbidity [58–60]. High turbidity can indicate containing higher concentration of pathogen in the water [9], high runoff intensity, and effluent discharge. This study could not analyse the correlation between* Cyclospora* and the physicochemical parameters because the number of positive samples were too small.

The limitation faced in this study was the small sample size that could be less significant to represent the whole population. We recommend further study to involve more samples, frequent sampling, and variety of station locations. Besides, species distribution of both parasites should be studied to predict the actual risk of infection by human-pathogenic genotypes via molecular techniques.

## 5. Conclusion

The findings of this study revealed that higher concentrations of* Cryptosporidium* than* Cyclospora* were found in water used for abstraction of drinking water treatment plant and recreational activities in Sarawak, Malaysia.

## Figures and Tables

**Table 1:** Concentration of *Cryptosporidium* and *Cyclospora* oocysts from water samples abstracted by drinking water treatment plants.

Water sample	Date	Station	Water column	Oocyst/L^a^	
				*Cryptosporidium*	*Cyclospora*
Sungai Sarawak Kiri	25-01-17	Downstream	Surface	0.9	0.2
			Subsurface	1.1	0.1
		Midstream	Surface	0.3	ND
			Subsurface	0.2	ND
		Upstream	Surface	0.2	ND
			Subsurface	ND	ND
	08-02-17	Downstream	Surface	1.0	ND
			Subsurface	2.7	ND
		Midstream	Surface	0.4	ND
			Subsurface	0.7	ND
		Upstream	Surface	0.3	ND
			Subsurface	ND	ND

Total positive sample				10/12 (83.33%)	2/12 (16.67%)

Sungai Sarawak Kanan	01-02-17	Downstream	Surface	ND	ND
			Subsurface	ND	ND
		Midstream	Surface	0.3	ND
			Subsurface	ND	ND
		Upstream	Surface	0.5	ND
			Subsurface	ND	ND
	27-02-17	Downstream	Surface	ND	ND
			Subsurface	ND	ND
		Midstream	Surface	ND	ND
			Subsurface	0.1	ND
		Upstream	Surface	0.8	ND
			Subsurface	ND	ND

Total positive sample				4/12 (33.33%)	0/12 (0.00%)

^a^Oocyst/L denotes that the number of the oocysts is expressed per litre of water sample; ND denotes not determined.

**Table 2:** Mean concentration of *Cryptosporidium* and *Cyclospora* oocysts according to sampling sites of the river system.

River stream	*Cryptosporidium* Oocyst/L (mean ± SD)	*Cyclospora* Oocyst/L (mean ± SD)
Upstream	0.22 ± 0.29	0.0 ± 0.0
	*N* = 4	*N *= 4
Midstream	0.23	0.0 ± 0.0
	*N* = 4	*N *= 4
Downstream	**0.71 ± 0.94**	**0.03 ± 0.07**
	**N = 4**	**N = 4**

*N* signifies total number of the samples.

**Table 3:** Mean concentration of *Cryptosporidium* and *Cyclospora* oocysts according to surface and subsurface river level.

Water column	*Cryptosporidium *	*Cyclospora *
	Oocyst/L	Oocyst/L
	(mean ± SD)	(mean ± SD)
Surface	0.391 ± 0.347	**0.016 ± 0.057**
	*N* = 12	**N = 12**
Subsurface	**0.4 ± 0.804**	0.008 ± 0.028
	**N = 12**	*N* = 12

*N* signifies the total number of samples.

**Table 4:** Concentration of *Cryptosporidium* and *Cyclospora* oocysts from water samples for recreational activities.

Water sample	Date	Station	Oocyst/L^a^	
			*Cryptosporidium*	*Cyclospora*
UNIMAS Lake (West Campus)	15-02-17	Outlet	ND	ND
		Inlet	0.2	ND
		Inlet	0.7	ND
	13-03-17	Outlet	ND	ND
		Inlet	1.2	0.2
		Inlet	0.7	0.6

Total positive sample			4/6 (66.67%)	2/6 (33.33%)

Ranchan Recreational Park	15-02-17	Downstream	ND	ND
		Midstream	ND	ND
		Upstream	ND	ND
	13-03-17	Downstream	ND	ND
		Midstream	ND	ND
		Upstream	ND	ND

Total positive sample			0/6 (0.00%)	0/6 (0.00%)

^a^Oocyst/L denotes that the number of the oocysts is expressed per litre of water sample; ND denotes not determined.

**Table 5:** Pearson correlation *r* and *p* values for *Cryptosporidium* and *Cyclospora* tested for bivariate correlation with temperature, pH, turbidity, dissolved oxygen, conductivity, and Total Dissolved Solid.

Parasite	Correlation of oocyst with physicochemical parameters					
	Temperature	pH	Turbidity	Dissolved oxygen	Conductivity	Total Dissolved Solid
*Cryptosporidium*	*r* = 0.321 *p* = 0.056	*r* = −0.124 *p* = 0.470	*r* = 0.042 *p* = 0.808	*r* = −0.434^*∗∗*^ *p* = 0.008	*r* = 0.123 *p* = 0.476	*r* = 0.238 *p* = 0.162
*Cyclospora*	*r* = 0.517^*∗∗*^ *p* = 0.001	*r* = 0.010 *p* = 0.954	*r* = 0.013 *p* = 0.941	*r* = −0.395^*∗*^ *p* = 0.017	*r* = 0.065 *p* = 0.708	*r* = 0.152 *p* = 0.375

*r* value signifies correlation coefficient value; *p* value signifies probability value. ^*∗∗*^Correlation is significant at the 0.01 level (2-tailed). ^*∗*^Correlation is significant at the 0.05 level (2-tailed).
